# Maternal and Fetal Outcomes of Pregnant Women with Hepatic Cirrhosis

**DOI:** 10.1155/2020/5819819

**Published:** 2020-01-08

**Authors:** Harun Egemen Tolunay, Mesut Aydın, Numan Cim, Barış Boza, Ahmet Cumhur Dulger, Recep Yıldızhan

**Affiliations:** ^1^Department of Obstetrics and Gynaecology, Van Yuzuncu Yil University, School of Medicine, Van, Turkey; ^2^Department of Gastroenterology, Van Yuzuncu Yil University, School of Medicine, Van, Turkey

## Abstract

**Aim:**

The reproductive hormone levels and systemic physiology of women with hepatic cirrhosis are altered. Existing data have indicated the adverse effects of cirrhosis on both the mother and the fetus. Pregnancy is successful in most of the patients with chronic liver disease. But maternal and fetal complication rates are still high for decompensated hepatic cirrhosis. In this study, we aimed to evaluate the clinical features, etiological factors, medications, morbidity, mortality, and obstetric outcomes of pregnant women with hepatic cirrhosis.

**Methods:**

Pregnant women, who were diagnosed with maternal hepatic cirrhosis and followed up in our clinic between 2014 and 2017, were retrospectively evaluated. The pregnant women that had been followed up for hepatic cirrhosis were classified as compensated disease and decompensated disease. Eleven cases were included in this period.

**Results:**

The mean age of cases was 33.5 ± 5.5 years. The mean gravida number was 3.2 ± 1.1, and the mean parity number was 1.7 ± 1. Six cases were in the compensated cirrhosis stage, and 5 cases were in the decompensated cirrhosis stage. A pregnancy with decompensated cirrhosis was terminated after the fetal heart sound was negative in the 9th week of pregnancy. Spontaneous abortus occurred in one case (<20 weeks). The mean gestational week of the 9 cases was 33.3 ± 6.2. Two of the 9 cases delivered birth vaginally. Seven cases delivered by cesarean section. The mean first- and fifth-minute APGAR scores were 6.6 ± 1.41 and 8.2 ± 1.56, respectively. The mean birth weight was 2303 ± 981 g. Among 9 cases with live birth, 6 had compensated cirrhosis and 3 had decompensated cirrhosis. In the second trimester, upper gastrointestinal endoscopy was performed to all patients in terms of esophageal varices. Endoscopic band ligation was performed in 3 cases with upper gastrointestinal bleeding. The postpartum mortality did not occur. *Discussion*. Pregnancy is not recommended for patients with hepatic cirrhosis due to high maternal and fetal morbidity and mortality. The pregnancy course of cases with cirrhosis changes according to the stage of liver injury and severity of disease. Although the delivery method is controversial, delivery by cesarean section is recommended for patients with esophageal varices by the reason of bleeding from varices after pushing during labor. The bleeding risk must be kept in mind as coagulopathy is common in hepatic diseases. The maternal-fetal morbidity and mortality rates have been decreased by the current developments in hepatology, prevention of bleeding from varices with drugs and/or band ligation, improvement in liver transplantation, and increasing experience in this issue.

## 1. Introduction

Cirrhosis which is characterized by generalized fibrosis and regenerative nodules is the end stage of hepatic parenchymal diseases. The prevalence of cirrhosis in women at the reproductive period has been reported 0.45/1000 [1]. The main reason for the rarity of cirrhosis that complicated pregnancy is that cirrhosis occurs after the reproductive period and cirrhosis-related metabolic abnormalities lead to anovulation and infertility [2]. For this reason, the real incidence of cirrhosis in pregnancy is unknown. The main etiology is postinfectious cirrhosis secondary to hepatitis B and hepatitis C virus infections in pregnancy. Icterus, edema, coagulopathy, metabolic abnormalities, portal hypertension, and splenomegaly are the most common clinical findings in these patients. Women with symptomatic cirrhosis are generally infertile. Chronic hyperestrogenemia and anovulation due to impaired estrogen metabolism after hepatocyte injury adversely affect fertility. The risk of serious complications such as hepatic failure, bleeding from varices, hepatic encephalopathy, splenic vein rupture, spontaneous abortus, preterm delivery, intrauterine growth failure, maternal and fetal mortality is increased during pregnancy. It is reported that especially patients with esophageal varices and severe portal hypertension have poor prognosis [3, 4]. Thus, the close follow-up of pregnancy, early recognition, and appropriate management of complications are important. In this study, fetal and maternal outcomes of pregnant women with hepatic cirrhosis in a tertiary health center were discussed in company with the literature.

## 2. Materials and Methods

Patients with hepatic cirrhosis that were followed in a tertiary health center between 2014 and 2017 were evaluated retrospectively. There was no pregnant woman with liver transplantation. Pregnant women with hepatic cirrhosis were classified as compensated and decompensated. In this period, 11 new cases were included. The ethical approval was not taken as the study was based on the evaluation of retrospective records.

The follow-up of patients was performed by gastroenterology and perinatology departments. The clinic of gastroenterology made the laboratory exams including hemogram, INR, albumin, and bilirubin level measurements and followed the patients clinically for the presence of acid and encephalopathy. Upper gastrointestinal endoscopy was performed to all cases in the second trimester about esophageal varices. Concurrently, the biophysical evaluation of fetus and follow-up were performed by ultrasound. Required platelet transfusion and fresh frozen plasma replacement were performed before vaginal delivery or cesarean section.

## 3. Results

The mean age of cases was 33.5 ± 5.5 years. The mean gravida number was 3.2 ± 1.1, and the mean parity number was 1.7 ± 1. Six cases were in compensated stage, and 5 cases were in the decompensated stage. A case with decompensated cirrhosis was terminated at the 9th week, and spontaneous abortus occurred in another case with decompensated cirrhosis (<20 weeks). The mean gestational week of the 9 cases was 33.3 ± 6.2. Among 9 cases, vaginal delivery was performed in 2 cases and the cesarean section was performed in 7 cases. In the compensated cases, cesarean section was performed in 4 cases and vaginal delivery occurred in 2 cases. Cesarean section was performed in all 3 cases with decompensated cirrhosis. The mean first- and fifth-minute APGAR scores were 6.6 ± 1.41 and 8.2 ± 1.56, respectively. The mean birth weight was 2303 ± 981 g. Pregnancies with compensated cirrhosis ended up with live births of the 30th and upper gestational weeks. Cases with decompensated cirrhosis delivered on the 30th week and lower. The cesarean section occurred in 3 cases with decompensated cirrhosis at the 26th, 28th, and 28th week, respectively. Upper gastrointestinal bleeding was revealed in these 3 cases, and endoscopic band ligation was performed for all. There was no antepartum and postpartum maternal mortality (Table 1).

## 4. Discussion

In this study, 5 of 11 cases with hepatic cirrhosis were decompensated and pregnancy did not last in 2 of these cases. The pregnancy was terminated in a case due to negative fetal heart sounds. Spontaneous abortus before the 20th week was revealed in another case. The 3 decompensated cases became complicated. Maternal and fetal deterioration was seen in these cases. All of these 3 cases with decompensated cirrhosis had upper gastrointestinal bleeding and delivered by cesarean section before the 30th week. The cesarean section is recommended for patients with esophageal varices due to bleeding from varices during pushing in vaginal delivery. In cases with compensated cirrhosis, delivery occurred at the 30th week and above. The outcomes of pregnancies with compensated cirrhosis were better.

The decompensation is the development of one or more findings which are acid, bleeding from varices, hepatic encephalopathy, and icterus. Among these, the most common finding is acid [5]. In our study, the common finding of 5 decompensated cases is acid.

The maternal-fetal effects of cirrhosis and portal hypertension were bleeding from esophagus varices, hepatic decompensation, acid, spontaneous bacterial peritonitis, hepatic encephalopathy, splenic artery aneurysm rupture, postpartum bleeding, gestational hypertension, abruption placentae, stillbirth, and intrauterine growth retardation [6, 7]. Spontaneous abortus is more frequent in pregnant women with cirrhosis than the general population (30-40% vs. 15-20%, respectively) [8]. The ratio of prematurity in delivery of pregnancy with cirrhosis is two to four times more than the general population [6, 9]. Meanwhile, the mean perinatal mortality rate was 18% in previous years [10]. It has been decreased to 6% in recent years. Close monitorization of patients is important because the fetal mortality rate of pregnant women with decompensated cirrhosis is 12% [9].

The risk of spontaneous abortus, preterm delivery, postpartum bleeding, and perinatal mortality significantly increases in pregnant women with cirrhosis [6]. In the review of MacDorman et al., the risk of perinatal mortality was reported 18% in maternal cirrhosis and the most important cause of mortality was premature labor [11]. Similarly, prematurity was indicated as a significant issue in our study. According to the previous data, spontaneous abortus related to decompensated cirrhosis occurred in one case.

Our study is similar to the literature in terms of the other obstetric complications of pregnant women with cirrhosis. There was no significant difference between pregnant women with cirrhosis and without cirrhosis, for the aspect of other obstetric complications such as gestational diabetes, infections, and thromboembolism, like other data in the literature [2]. Bleeding from varices develops most frequently in the second and third trimester when blood volume reaches the maximum and the pressure of the fetus above abdominal vessels is more distinct. Tan et al. reported that 62% of the bleeding from varices developed at 20-26th gestational week when the volume increment was significant [6]. Although sclerotherapy as an eligible treatment is a promising alternative in the early years, it has been yielded to endoscopic band ligation. Despite no prospective randomized study about this issue, most of the authors suggested that the band ligation had to be preferred to sclerotherapy due to its potential adverse effects of chemicals on pregnant [12]. Upper gastrointestinal endoscopy can be performed safely in pregnant woman, and the most important risk is fetal distress related to sedative drugs and the position of the patient. Though it is not evidence-based, most of the authors suggested endoscopy before pregnancy and the second trimester [6]. There is still no sufficient data about prophylactic endoscopic band ligation. As we reported, to provide against the bleeding at the second trimester is important for pregnant women with cirrhosis and esophagus varices. We performed endoscopy routinely in the second trimester. According to the literature, we treated bleeding from esophageal varices with therapeutic band ligation.

Beta-blocker drugs (propranolol and nadolol) that are used for the primary prophylaxis of bleeding from varices are categorized as Class C in pregnancy. The advantage of these drugs predominates the adverse effects in pregnant women with high risk of bleeding. The most common morbidity of these drugs is fetal growth retardation, hypoglycemia, and neonatal bradycardia [6, 13].

In their study that included 117 pregnant women with cirrhosis secondary to various etiologies, Cheng et al. reported that 44.4% of patients had impairment in liver functions during pregnancy; 21.3% of patients had hematemesis. The authors suggested that these pregnancies were managed properly and had good maternal outcomes [13, 14].

In conclusion, the subject to pregnant women with cirrhosis currently increases via the improvement of chronic liver disease treatment. It should be kept in mind that the risk of metabolic, obstetric, and perinatal morbidity and mortality significantly increases in these patients. Follow-up of pregnant women with cirrhosis by a multidisciplinary team including gastroenterology, general surgery, perinatology, and pediatrics is vital for the management of complications at the appropriate time.

## Figures and Tables

**Table 1:** Clinical features of patients.

Case no.	Age	Gravida	Parity	Delivery method	Birth week	Birth weight (gram)	APGAR score 1 min	APGAR score 5 min	Compensation	Upper gastrointestinal bleeding
1	28	3	2	Cesarean	26	980	4	6	Decompensation	Yes
2	33	5	4	Cesarean	42	3400	8	10	Compensation	No
3	28	4	1	Spontaneous abortion	<20	<500	Ø	Ø	Decompensation	No
4	42	2	1	Pregnancy termination	<20	<500	Ø	Ø	Decompensation	No
5	33	4	2	Cesarean	28	1420	6	8	Decompensation	Yes
6	24	3	2	Cesarean	30	1900	6	8	Compensation	No
7	36	5	3	Cesarean	38	3150	8	8	Compensation	No
8	41	3	1	Cesarean	42	3700	8	10	Compensation	No
9	38	3	1	Vaginal delivery	36	2850	8	10	Compensation	No
10	32	2	1	Cesarean	28	1540	6	8	Decompensation	Yes
11	34	2	1	Vaginal delivery	30	1790	6	6	Compensation	No

## Data Availability

The [DATA TYPE] data used to support the findings of this study are included within the article.
